# New Appliances and Things Medical

**Published:** 1905-11-25

**Authors:** 


### NEW APPLIANCES AND THINGS MEDICAL.

[We shall be glad to receive, at our Office, 28 & 29 Southampton Street, Strand, London, W.C., from the manufacturers, specimens of all new preparations and appliances which may be brought out from time to time.]

### "CAWARRA" AUSTRALIAN WINES.

(H. J. Lindeman, Sydney, N.S.W., and Arthur Marshall & Sons, 7 East India Avenue, London, E.C.)

We have received samples of these wines—namely, claret, hock, Burgundy, Muscat, and Chablis. The wines are all of good flavour and closely approximate in alcoholic strength to the European wines of the same name. The Burgundy and Muscat are heavier and of fuller flavour than the others, and can both be described as sound wines of a pleasant character. The claret, hock, and Chablis go well with mineral-water, and in this form make an exceedingly pleasant drink. We regard this feature as an important one, in that in this country the drinking of wines with water or mineral-water is not nearly so common as in France, and the wine-growing countries generally. Gastronomically the dilution of wines with such a water is advisable. We think the Australian wines before us are distinctly to be recommended and likely to have an extensive sphere of usefulness.

### SCOTCH WHISKEY.

(James Watson & Co., Ltd., Seagate, Dundee.)

We are in receipt of two samples of Scotch whiskey from this well-known firm. The first sample is "Blue Riband Glenlivet Three Star Whiskey." This spirit is a typical good Scotch whiskey, having a full and pleasant flavour and taste. The whiskey, as is the case with the vast majority of whiskeys, is blended—*i.e.* is a mixture of whiskeys, containing no doubt spirit derived from both the pot and patent still. The second sample before us is termed No. 10, and is described on the label as very fine old Scotch whiskey. This spirit has also a good flavour and taste, and possesses all the characteristics of a sound whiskey. We should have no hesitation in recommending either of these two spirits to patients in whom the use of whiskey was indicated, as they both show the presence, chemically as well as physiologically, of the by-products of ethyl alcohol, to which, together with the latter substance, the specific action of whiskey is due.

